# Pathway activity inference for multiclass disease classification through a mathematical programming optimisation framework

**DOI:** 10.1186/s12859-014-0390-2

**Published:** 2014-12-05

**Authors:** Lingjian Yang, Chrysanthi Ainali, Sophia Tsoka, Lazaros G Papageorgiou

**Affiliations:** Centre for Process Systems Engineering, Department of Chemical Engineering, University College London, London, WC1E 7JE UK; Department of Informatics, School of Natural and Mathematical Sciences, King’s College London, London, WC2R 2LS UK

**Keywords:** Disease classification, Microarray, Pathway activity, Mathematical programming, Optimisation

## Abstract

**Background:**

Applying machine learning methods on microarray gene expression profiles for disease classification problems is a popular method to derive biomarkers, i.e. sets of genes that can predict disease state or outcome. Traditional approaches where expression of genes were treated independently suffer from low prediction accuracy and difficulty of biological interpretation. Current research efforts focus on integrating information on protein interactions through biochemical pathway datasets with expression profiles to propose pathway-based classifiers that can enhance disease diagnosis and prognosis. As most of the pathway activity inference methods in literature are either unsupervised or applied on two-class datasets, there is good scope to address such limitations by proposing novel methodologies.

**Results:**

A supervised multiclass pathway activity inference method using optimisation techniques is reported. For each pathway expression dataset, patterns of its constituent genes are summarised into one composite feature, termed pathway activity, and a novel mathematical programming model is proposed to infer this feature as a weighted linear summation of expression of its constituent genes. Gene weights are determined by the optimisation model, in a way that the resulting pathway activity has the optimal discriminative power with regards to disease phenotypes. Classification is then performed on the resulting low-dimensional pathway activity profile.

**Conclusions:**

The model was evaluated through a variety of published gene expression profiles that cover different types of disease. We show that not only does it improve classification accuracy, but it can also perform well in multiclass disease datasets, a limitation of other approaches from the literature. Desirable features of the model include the ability to control the maximum number of genes that may participate in determining pathway activity, which may be pre-specified by the user. Overall, this work highlights the potential of building pathway-based multi-phenotype classifiers for accurate disease diagnosis and prognosis problems.

**Electronic supplementary material:**

The online version of this article (doi:10.1186/s12859-014-0390-2) contains supplementary material, which is available to authorized users.

## Background

The popularity of microarray technology as means of deriving a comprehensive view of gene expression under particular environmental stimuli has necessitated the development of computational strategies for linking expression patterns to sample phenotypes [1,2]. In charactering disease, the gene expression matrix serves as input to a classification task where each sample is allocated to a relevant phenotypic class via specific gene signatures or biomarkers that can best differentiate between outcomes. Such disease classification tasks have been successful in deriving biomarkers for diagnosis [3], prognosis [4-7] and response to treatment [8,9] in complex disorders.

Despite successful reports, disease classification is impeded by the so-called “large p small n” property, whereby the number of samples (or instances) is typically several orders of magnitude smaller than the number of genes (or features), making it difficult to extract reliable information from transcriptomic profiles [10]. Feature reduction methods are therefore employed as means of extracting a smaller set of genes, able to discriminate between disease states. We note as example methods based on partial least squares [11], heuristic breadth-first search algorithm [12], and ensemble feature selection methods [13]. Subsequently, a classifier can be trained on the reduced feature set to predict the disease status or prognostic characteristic of any given samples [14-17].

Such diagnostic or prognostic profiles relate to genes that do not act in isolation, but in fact work in concert, forming sub-networks that collectively modulate or determine cell fate. Accounting for such molecular synergies in feature reduction and disease classification protocols can also alleviate challenges of single-gene classifiers related to cellular heterogeneity in tissue, genetic heterogeneity among patients, measurement noise [18-20], thereby leading to increased biological interpretability of biomarkers and enhancing insights into the mechanisms of the disease [21-23]. Therefore, feature selection and classification methods where all genes are treated independently are increasingly replaced by approaches where the effects of *groups* of genes on disease prediction are considered simultaneously. Such gene sets can either reflect curated biochemical pathways or functional modules derived from protein interaction networks [19,24-34].

The availability of pathway information from publc databases, for example Kyoto Encyclopedia of Genes and Genomes (KEGG) [35], Gene Ontology (GO) [36] and Reactome [37], provide the possibility of analysing functional sets of genes that fall within common pathways and identifying the disease-relevant pathways as biomarkers. Initial efforts of gene-set based approaches included gene set enrichment analysis [38], which calculates to what extent a set of genes show statistically significant difference between samples belonging to either of the two phenotypes. Other similar computational tools have also been reported [39-44]. However, those statistical frameworks commonly assign one score for each set of genes to quantify the deregulation of this gene-set under disease status of interest, but do not provide more information on the level of gene-set deregulation for each sample. It is argued that this drawback compromises their potential in personalised pathway analysis [26].

Therefore, a more informative approach may be to assign a score to each pathway and sample, which represents the activity of that particular pathway for that sample [19,25-28,45-47]. The mean and median expression value across all constituent genes within a pathway, termed *pathway activity*, has been proposed in [28]. Other studies produce pathway activity measures based on principle component analysis (PCA) to derive the top principle component that captures the maximum variance in the dataset [26,45,48,49]. More recently a supervised greedy search algorithm was proposed that ranks genes according to their individual discriminative power and then searches for a subset of highly ranked genes whose averaged expression profiles yield better discriminative power [19]. This method was modified so that it accounts for up- and down-regulated genes by assigning positive sign and negative sign respectively [27]. Both methods are inherently applicable to binary classification problems. A statistical inference method [25] proposes to aggregate the probabilistic evidence of all genes within a pathway for predicting a sample into one of the two phenotypes. Other relevant studies based on the concept of pathway activity either require other biological information as prior, for example copy number variation and protein interactions [20,47,50] or are not designed for classification tasks [47,51].

Pathway activity-based classification approaches provide competitive or higher prediction accuracy when compared to traditional single genes-based classifiers [19,52], so extending or refining their use is a promising avenue for biomarker discovery. Despite rapidly increasing interest in developing novel and robust pathway activity inference methods, most of the existing methods still use rather simple means of summarising the expression patterns of either some or all constituent genes into the composite pathway level attribute, for example the mean or median value of sample expression across all or a subset of constituent genes [19,28]. PCA-based methods [26,45,48,49] calculate the first principal component, representing the maximum variance of the data set, as pathway activity. However such methods do not take into account the phenotype information of samples. Furthermore, some current pathway activity inference methods are constrained to two-phenotype (binary) classification problems [19,20,25,27], disallowing their use in more complex problems of multi-phenotype classification.

In this work, we propose a novel multiclass method that infers pathway activity in a supervised manner. The proposed method summarises expression patterns of constituent genes into pathway activity via weighted linear summation of gene expression. As opposed to some methods in literature where gene weights are taken as a prior, in our work gene weights are decided by the model, so that the constructed pathway activity can optimally distinguish samples from different phenotypes. Furthermore, the mathematical framework of this method offers the ability to the user to explicitly constraint the maximum number of constituent genes contributing to pathway activity inference. Using a number of published gene expression profile datasets, we show that this pathway activity inference method is robust in terms of the number of constituent genes allowed to determine the pathway activity metric. Comparative analyses show that the method is an effective means of reducing classification features, as it either outperforms or at least matches competing pathway activity inference methods in two-phenotype disease classification problems, and provides significantly better classification rates in multi-phenotype classification problems.

## Methods

### Data sources

Complex diseases such as breast cancer and psoriasis are the product of multiple gene interactions that collectively contribute to the etiology of the disease through largely unknown mechanisms [53]. Breast cancer is the most frequently diagnosed malignancy and has been intensively studied by gene expression profiling [4-6,54-57]. Psoriasis is a systemic, inflammatory skin disease with autoimmune underpinnings affecting 2-3% of the world population [58-60]. Prostate tumor is the most frequently diagnosed cancer in American men [61] and displays a broad range of clinical and histological behaviors [3,62]. Diffuse large B-cell lymphoma (DLBCL) is the most common lymphoid malignancy in adults [63] with less than 40% patients responding desirably to the current therapy while the remainders succumb to the disease, highlighting the unidentified molecular heterogeneity in the tumors [64].

A total number of 8 published microarray gene expression profiles were obtained that represent these diseases (Table 1). In terms of disease phenotypes in these datasets, used as class outcomes in the relevant classification tasks, for psoriasis samples are either lesional or non-lesional tissue from psoriasis patients, as well as healthy controls [65,66]. For breast cancer, 49 samples belong to three disease classes, apocrine, basal and luminal [67]; 139 samples are divided into healthy, luminal, ERBB2 and basal [68]; expression profiles of 230 breast cancer patients 48 of whom became residual invasive cancer free in the breast or lymph nodes after a 6-month preoperative chemotherapy and the remainder still had residual invasive cancer after the treatment. Gene expression data were generated using specimens of breast cancer before any treatment [8]; lymph-node negative breast cancer patients with some of them diagnosed with distant metastasis [4]. For prostate cancer, 102 expression profiles are used to distinguish tumour samples from normal samples [3]. Finally, 77 expression profiles of patients either diagnosed with diffuse large B-cell lymphoma or follicular lymphoma (FL) are used [63].

**Table 1:** **Datasets**

**Dataset**	**Disease**	**Samples**	**Samples per phenotype**	**Source**
Swindell [65]	Psoriasis	180	Healthy control: 64;	GSE13355
			Psoriatic non-lesional skin: 58;	
			Psoriatic lesional skin: 58	
Yao [66]	Psoriasis	82	Healthy control: 21;	GSE14905
			Psoriatic non-lesional skin: 28;	
			Psoriatic lesional skin: 33	
Farmer [67]	Breast cancer	49	Apocrine tumour: 6;	GSE1561
			Basal tumour: 16;	
			Luminal tumour: 27	
Pawitan [68]	Breast cancer	139	Normal: 37;	GSE1456
			Luminal tumour: 62;	
			ERBB2: 15;	
			Basal: 25	
Singh [3]	Prostate cancer	102	Normal: 50;	www.broadinstitute.org
			Tumour: 52	
Shipp [63]	DLBCL	77	DLBCL: 58;	www.broadinstitute.org
			Follicular lymphoma: 19	
Popovici [8]	Breast cancer	230	Residual invasive cancer: 182;	GSE24061
			No residual invasive cancer: 48	
Desmedt [4]	Breast cancer	198	Metastatic: 51;	GSE7390
			Non-metastatic: 147	

All microarray datasets have been obtained on Affymetrix platforms. For each dataset, raw data have been downloaded and pre-processed using the Bioconductor package LIMMA [69]. KEGG C2 functional gene sets have been downloaded from MsigDB database (v3.0, Sep 2010) [70], which included a total number of 186 curated pathways and 5267 genes.

### Pathway activity-based classification procedure

**Figure 1:**
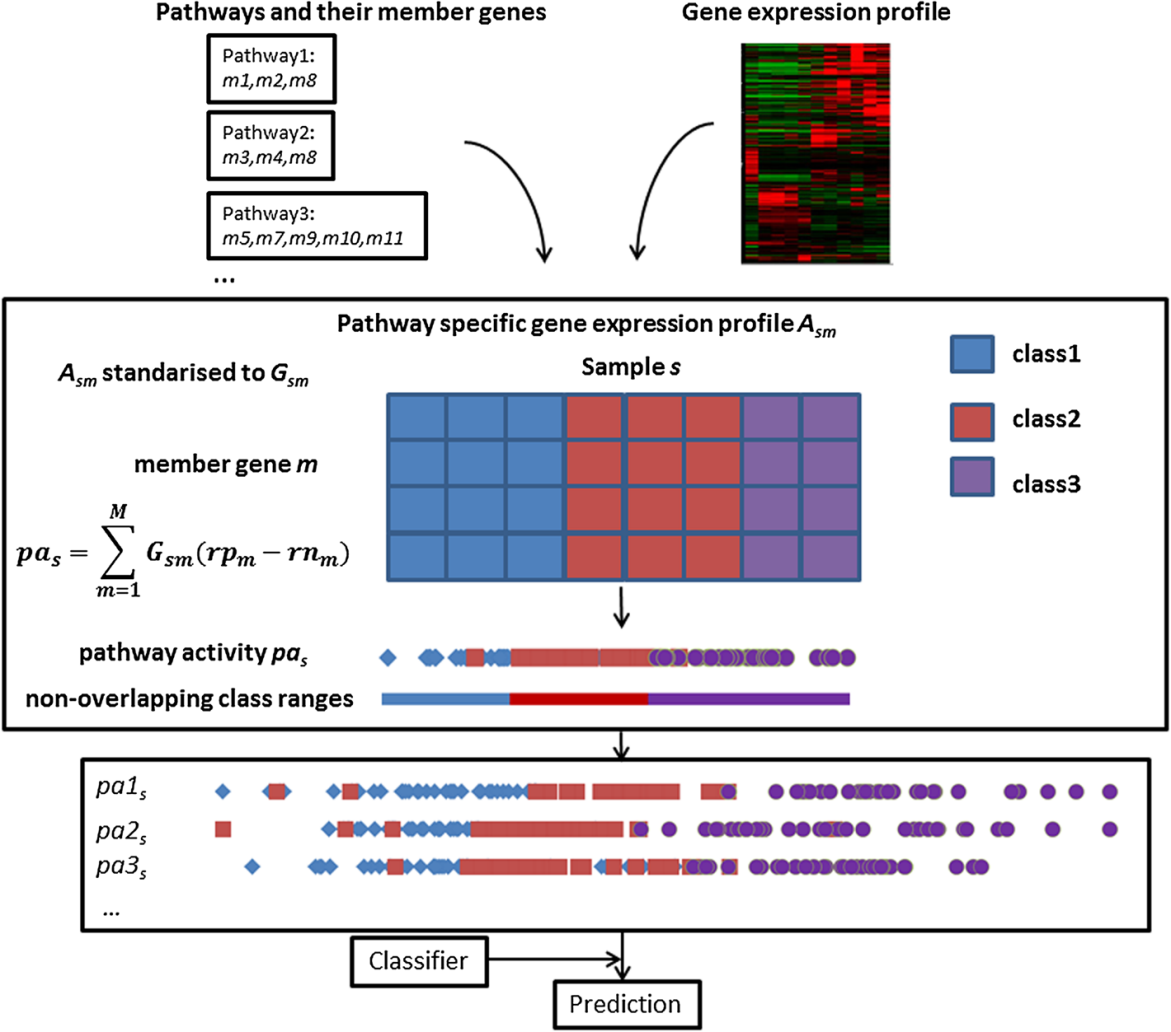
**Schematic flow chart of the DIGS-based approach for multiclass disease classification problems.** Pathway specific gene expression profiles are created by integrating gene expression profile and pathway information. For each pathway, build pathway activity as a weighted (variables) linear summation of expression of member genes, with the objective function maximising the number of samples whose pathway activity are inside the range of their own classes. The maximum number of member genes in a pathway allowed to have non-zero weights is explicitly constrained in the model by specifying the parameter *NoG*. Create pathway activity profile by assembling all pathway activities and a classifier is trained on the pathway activity profile and predicts the class label of a new sample. It is important to note that training procedure, i.e., inferring pathway activity and training a classifier, is always blind to testing samples to achieve an objective evaluation of classification performance.

An overview of the computational procedure developed for pathway activity-based disease classification is illustrated in Figure 1. A microarray gene expression profile and a set of pathways with their constituent genes form the input to create pathway-specific gene expression matrices. For each pathway, *m* denotes member genes, *s* samples and *A*_*sm*_ the expression value of gene *m* in sample *s. A*_*sm*_ are standardised to *G*_*sm*_ by subtracting the population mean from the raw value and then dividing by the standard deviation. The first stage of our computational procedure derives a new composite feature, pathway activity *pa*_*s*_, from the standardised pathway specific gene expression profile *G*_*sm*_. In the second stage of our protocol, the inferred pathway activities for all pathways are assembled to form a pathway activity profile matrix, on where a classifier is trained to predict the phenotype of a new sample. In next section, we present a novel mathematical model, which infers pathway activity with optimal classification accuracy.

### A novel mathematical programming formulation to infer pathway activity

The indices, parameters and variables used in the model to infer pathway activity for each pathway are described here and are listed in Additional file 1. Two sets of positive variables *rp*_*m*_ and *rn*_*m*_ are introduced, quantifying the positive and negative weights of gene *m* towards pathway activity inference. For sample *s,* pathway activity, *pa*_*s*_, is defined as the summation of the standardised gene expression values, *G*_*sm*_ multiplied by the gene weight (*rp*_*m*_-*rn*_*m*_) over all member genes:

1 $$ p{a}_s={\displaystyle \sum_{m=1}^M}{G}_{sm}\left(r{p}_m-r{n}_m\right)\kern2.5em \forall s=1,2,\dots S $$

where *M* is the total number of member genes for this particular pathway and *S* is the total number of samples. Both positive and negative weights of a gene *m* are defined as positive continuous variables; their values are determined by the optimisation model. One set of binary variables, *L*_*m*_, which takes values of either *0* or *1* has been introduced, while equations (2) and (3) below ensure that for each gene *m at most* one of *rp*_*m*_ and *rn*_*m*_ can take positive values:

2 $$ r{p}_m\le {L}_m\kern2.5em \forall m=1,2,\dots, M $$

3 $$ r{n}_m\le 1-{L}_m\kern1em \forall m=1,2,\dots, M $$

When *L*_*m*_ *= 1*, *rp*_*m*_ can take any value between *0* and *1* while *rn*_*m*_ is forced to be equal to *0*; otherwise when *L*_*m*_ *= 0*, *rp*_*m*_ is forced to be equal to *0* while *rn*_*m*_ can be between *0* and *1*. In either case, both *rp*_*m*_ and *rn*_*m*_ can be equal to *0*, which means this particular gene has zero weight in inferring pathway activity. Overall, a gene can have positive, negative or zero weight towards the composite feature construction. For normalisation purpose, the summation of absolute gene weights should be equal to one:

4 $$ {\displaystyle \sum_{m=1}^M}\left(r{p}_m+r{n}_m\right)=1 $$

Inspired by Lee et al. [19], where a small subset of member genes is selected (usually less than 7) to construct pathway activity, we add constraints to limit the number of genes having non-zero weights in inferring pathway activity. Thus a new set of binary variables, *W*_*m*_, are introduced to the model to indicate whether a member gene *m* is active, i.e. having non-zero weights in constructing pathway activity or not:

5 $$ r{p}_m+r{n}_m\le {W}_m\kern2em \forall m=1,2,\dots, M $$

If *W*_*m*_ takes the value of *0* then both positive weight (*rp*_*m*_) and negative weight (*rn*_*m*_) of gene *m* are forced to be equal to *0*, while when *W*_*m*_ is equal to *1*, gene *m* is allowed to take any weight (*rp*_*m*_*-rn*_*m*_) between −*1* and *1*. The next equation restricts the maximum number of genes allowed to have non-zero weights to a manually specified value (*NoG*):

6 $$ {\displaystyle \sum_{m=1}^M}{W}_m\le NoG $$

In the case where *NoG* is equal to or larger than the number of member genes available in the pathway, the constraint is redundant as all the member genes will be allowed to take any weight (*rp*_*m*_*-rn*_*m*_) between −*1* and *1*. We aim to construct pathway activity as a feature with good discriminative power, which can separate samples from different phenotypes as much as possible.

For each phenotype/class *c*, two continuous variables have been introduced as *LO*_*c*_ and *UP*_*c*_, denoting the lower and upper bound respectively, of the range of pathway activity for phenotype *c*. In addition, a set of binary variables, *E*_*s*_*,* have been introduced, defined by:

$$ {E}_s=\left\{\begin{array}{l}1,\kern0.15em  if\  activity\  value\  of\  sample\ s\  falls\  within\  the\  lower\  and\  upper\  bounds\  of\\ {}\mathrm{its}\ \mathrm{class}\ \mathrm{range};\\ {}0,\ \mathrm{otherwise}\end{array}\right. $$

together with the following constraints:

7 $$ 0\le p{a}_s-L{O}_c+U\left(1-{E}_s\right)\kern2em \forall s=1,2,\dots, S,{c}_s $$

8 $$ {\mathrm{pa}}_s-U{P}_c-U\left(1-{E}_s\right)\le 0\kern2em \forall s=1,2,\dots, S,{c}_s $$

where *c*_*s*_ is the phenotype for sample *s* and *U* is an arbitrarily large positive number. On the constructed pathway activity, ranges of different classes are not allowed to overlap. A set of binary variables, *Y*_*kc*_, have been introduced as follows:

$$ {Y}_{kc}=\left\{\begin{array}{l}1,\kern0.15em \mathrm{if}\ \mathrm{upper}\ \mathrm{bound}\ \mathrm{o}\mathrm{f}\ \mathrm{r}\mathrm{ange}\ \mathrm{f}\mathrm{o}\mathrm{r}\ \mathrm{c}\mathrm{lass}\ \mathrm{k}\ \mathrm{is}\ \mathrm{lower}\ \mathrm{than}\ \mathrm{lower}\ \mathrm{bound}\ \mathrm{o}\mathrm{f}\ \mathrm{r}\mathrm{ange}\\ {}\mathrm{f}\mathrm{o}\mathrm{r}\ \mathrm{c}\mathrm{lass}\ \mathrm{c}\ \mathrm{o}\mathrm{n}\ \mathrm{pathway}\ \mathrm{activity};\\ {}0,\ \mathrm{o}\mathrm{therwise}\end{array}\right. $$

The additional two sets of constraints have been introduced to guarantee the non-overlapping condition:

9 $$ U{P}_k+\varepsilon \le L{O}_c+U\left(1-{Y}_{kc}\right)\kern2.75em \forall k<c $$

10 $$ U{P}_c+\varepsilon \le L{O}_k+U{Y}_{kc}\kern5.5em \forall k<c $$

where *ε* is an arbitrarily small positive number ensuring that pair-wise classes do not share a border. Equations (9) and (10) are generated for each pair of classes. The objective of the optimisation problem is to infer the pathway activity such that it is as discriminative as possible, i.e. as many samples as possible can fall within the range of its corresponding classes (*E*_*s*_ *= 1*). In other words, the objective function is to minimise the number of misclassified samples:

11 $$ min{\displaystyle \sum_{s=1}^S}\left(1-{E}_s\right) $$

The resulting mathematical programming-based formulation for inferring pathway activity is summarised below:

- Objective function (11)
- Subject to:
- Pathway activity definition (1)
- Positive and negative gene effect constraints (2) and (3)
- Normalisation constraint (4)
- Restriction of the number of active genes (5) (6)
- Pathway activity enclosing constraints (7) and (8)
- Non-overlapping constraints for ranges of different classes (9) and (10)

$$ {L}_m,\kern0.15em {E}_s,\kern0.15em {W}_m,\kern0.15em {Y}_{kc}\in \left\{0,1\right\};\kern0.15em r{p}_m,\kern0.15em r{n}_m\ge 0;p{a}_s,\kern0.15em L{O}_c,\kern0.15em U{P}_c:\ \mathrm{unrestricted} $$

The proposed mathematical programming formulation consists of a linear objective function and a number of linear constraints. The linearity and presence of binary and continuous variables define a mixed integer linear programming (MILP) model, named DIGS (DIfferential Gene Signatures) here, and can be solved to global optimality using some of the standard algorithms like branch-and-bound.

To facilitate the use of DIGS, we make available the GAMS executable, example input files and user guide at www.ucl.ac.uk/~uceclap/DIGS.

### Comparison of the DIGS model with genes-based methods and other pathway activity inference methods

To compare the results obtained with the DIGS model, we have implemented a number of pathway activity methods from the literature (summarised in Table 2). In overview, these methods include: i) the method that uses the microarray gene expression profile without pathway information, for example SG; ii) the method that utilises pathway information but is based on the pathway specific gene expression profile instead of inferring pathway activity, for example per_pathway, and iii) those that take advantage of pathway information and infer pathway activity, for example [19,28,45].

**Table 2:** **Overview of Evaluated Methods**

Guo et al. [28]	**Abbreviation:** Mean
	**Computational basis:** Pathway activity
	**Description:** Create pathway-specific gene expression profiles; for each pathway, pathway activity for sample is its mean expression value among all member genes; a classifier is trained on pathway activity profile.
Guo et al. [28]	**Abbreviation:** Median
	**Computational basis:** Pathway activity
	**Description:** Create pathway-specific gene expression profiles; for each pathway, pathway activity for sample is its median expression value among all member genes; a classifier is trained on pathway activity profile.
Bild et al. [45]	**Abbreviation:** PCA
	**Computational basis:** Pathway activity
	**Description:** Create pathway-specific gene expression profiles; for each pathway, top principal component is calculated as the pathway activity; a classifier is trained on pathway activity profile.
Lee et al. [19]	**Abbreviation:** CORGs
	**Computational basis:** Pathway activity
	**Description:** Create pathway-specific gene expression profiles; for each pathway, apply *t*-test to rank genes and perform a greedy search to find a subset of genes whose averaged expression values is locally maximal in *t*-test value; a classifier is trained on pathway activity profile; only applicable for two-class problems.
Ainali et al. [72]	**Abbreviation:** Per_pathway
	**Computational basis:** Single genes
	**Description:** Create pathway-specific gene expression profiles; a classifier is trained on each pathway-specific gene expression profile separately, and prediction rates achieved by all pathway classifiers are averaged as the final prediction rate.
Single Genes	**Abbreviation:** SG
	**Computational basis:** Single genes
	**Description:** Apply [71] to select a subset of top genes; a classifier is trained on reduced gene expression profile
Proposed in this work	**Abbreviation:** DIGS
	**Computational basis:** Pathway activity
	**Description:** Create pathway-specific gene expression profiles; Apply the proposed DIGS model to construct pathway activity as weighted linear summation of gene expressions; a classifier is trained on pathway activity profile.

In detail, comparative results are presented by implementation of the following methods: i) a genes-based approach has been implemented for comparison where, given a whole gene expression profile, a feature selection [71] method is applied to select a subset of top genes with the best discriminative power for classification. The multiclass feature selection method [71] used here employs a distance metric, for example weighted *L*_*1*_ metric or K-L divergence and gives a subset of top attributes/genes with respect to the aggregated pair-wise class distances, where the number of attributes in the subset obtained is pre-set by the user. A classifier is then trained using only the small subset of discriminative genes for disease classification problems; ii) the Ainali et al. [72] method, where each pathway-specific gene expression profile is treated independently, i.e. training and testing are conducted for each pathway-specific expression profile separately and classification accuracies across all pathways are averaged to obtain the final classification rate (referred as *per_pathway*), and iii) the two methods from Guo et al. [28] (referred as *mean* and *median*, respectively), which take either the mean or median gene expression values of all genes within a pathway for each sample. The Bild et al. [45] approach (referred as *PCA*) of using the first principal component as representation of pathway activity, which represents a family of principal component analysis-based methods [26,48,49]. The Lee et al. [19] method, which identifies and averages a subset of condition-responsive genes (referred as *CORGs*), which has been implemented only for two-phenotype disease classification problems, as it is not suited to multi-class problems.

### Evaluation of classification performance

The performance of the various pathway activity metrics is evaluated by the classification accuracy achieved across the eight disease datasets. For each dataset, samples are split randomly in training and testing sets of 70 and 30% respectively and this procedure is repeated fifty times. Composite features are constructed using Mean, Median, CORGs, PCA and DIGS on the training samples, resulting in low dimensionality matrix of samples across pathway activities, on which five popular classifiers SMO [73], Neural Network (NN) [74], K-Nearest-Neighbours (K-NN) [75], Logistic Regression (Logistic) [76] and Hyperbox (HB) [77] are trained. The classifiers are then tested on the testing sample set and the prediction accuracy is calculated as the number of correctly classified samples divided by the total number of testing samples, averaged across the fifty training/testing sets.

The above procedure is modified where pathway activities are not used, i.e. in the SG and *per_pathway* approaches. In the genes-based approach, the feature selection method [71] has been applied using training samples only and the top genes are selected. The number of top genes is set to be identical to the number of pathways (i.e. 186) in order to derive comparable dimensionalities between the pathway activity-based and the genes-based approach. For the *per_pathway* approach, each of the 5 classifiers have been trained using training samples only and then validated on the testing samples sets for each pathway separately.

Overall, 8 microarray gene expression profiles (dataset), 7 competing methods (method) and 5 classifiers (classifier) are employed in our study. For each combination of dataset, method and classifier, classification accuracies over 50 individual testing sets are averaged as the prediction accuracy for this combination. It is important to note that Lee et al. [19] is applicable for only two-phenotype problems, therefore we divide the 8 datasets into a group of 4 binary datasets and the other group of 4 multiclass datasets. For the binary classification comparison, for each method we average the prediction accuracies over all 4 binary datasets and all 5 classifiers, which gives a comprehensive indication of the efficiency of the evaluated methods (i.e. Mean, Median, PCA, CORGs, per_pathway, SG and the proposed DIGS). For the multiclass case, the same analysis is applied and all comparative analyses are discussed in the next section.

The DIGS model has been implemented in the General Algebraic Modelling System (GAMS) [78] using the CPLEX MILP solver in a CentOS 5.2 64 bit Unix computer environment. The optimality gap is set as 0. Computational resource limit is set as 200 seconds per run. Among the 5 classifiers SMO, NN, K-NN and Logistic have been implemented in WEKA machine learning software [79] with the following parameters for NN: hidden layers 2, learning rate 0.1, momentum 0.2, training time 10000; and for K-NN: the number of nearest neighbours is selected as 5. For other classifiers, their default settings have been retained. HB has been reproduced in GAMS according to its original publication [77].

## Results and discussion

In this work, we propose an optimisation-based model that infers a pathway activity metric as a weighted linear combination of the constituent gene expression values. The DIGS model can identify a subset of pathway constituent genes with cardinality no more than the user-specified value, *NoG*, whose expression can be combined via different weights to best separate samples from different phenotypes. The effect of *NoG* is illustrated through sensitivity analysis below, followed by a comparison of the model against a variety of disease classification strategies, including both single-gene and pathway activity based approaches.

### Sensitivity analysis for *NoG,* influencing the number of active genes in constructing pathway activity

Parameter *NoG* determines the maximum number of pathway member genes that have non-zero weight in activity inference. Tuning this parameter is important as a small value may not fully utilise the discriminative member genes, while an excessively large value may potentially cause over-fitting, i.e. in the case where too many genes are allowed to take non-zero weights for pathway activity against a relatively small number of training samples, leading to decreased prediction accuracy.

**Figure 2:**
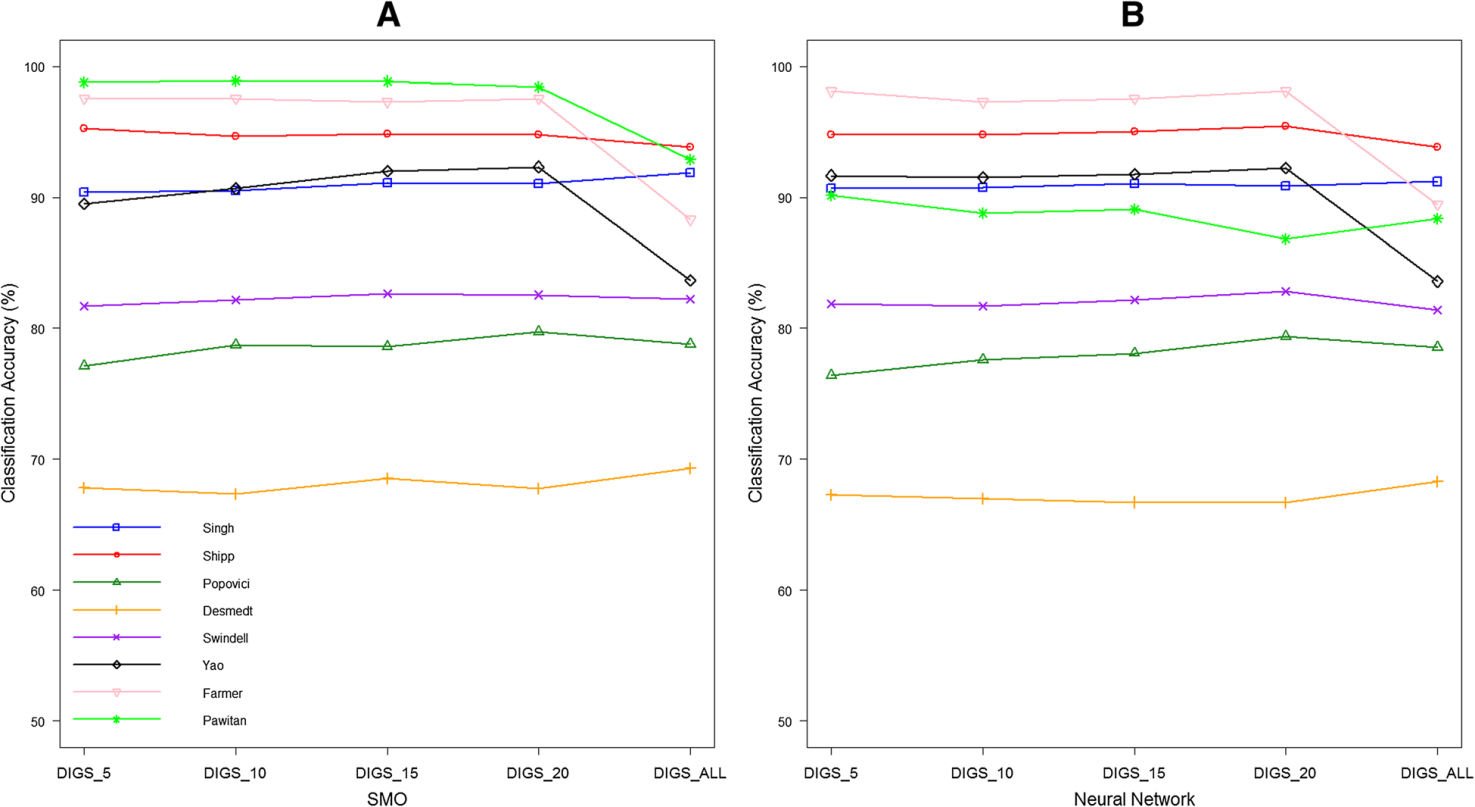
**Sensitivity analysis of parameter** ***NoG*** **for DIGS model with SMO (A) and NN (B) classifiers.** For each of the 8 datasets, the proposed DIGS model is applied to infer pathway activity while setting *NoG*, i.e. the maximum number of member genes in a pathway allowed to have non-zero weights, to *5, 10, 15* and *20*. In addition, DIGS model is also applied with *NoG* set to equal to the number of available member genes in a pathway, i.e. all member genes can take non-zero weights to construct pathway activity. A classifier is trained using the pathway activity profiles and tests the prediction accuracy. For both SMO **(A)** and NN **(B)** classifiers, it is clear that the proposed DIGS model is robust to the parameter *NoG* during the tested ranged *5* to *20*. Furthermore, constraining the maximum number of active constituent genes appears to generally improve classification accuracy as DIGS_ALL usually leads to lower prediction rate compared with the others.

Here, the DIGS model is applied to infer pathway activity with *NoG* set to *5, 10, 15* and *20,* followed by training and testing using a range of classifiers for each microarray dataset. As a comparison, DIGS is also run with *NoG* set equal to the number of member genes for each pathway, so as to allow *all* member genes in a pathway to take non-zero weights for pathway activity inference. The prediction rates achieved by these different values of *NoG* are denoted by DIGS_5, DIGS_10, DIGS_15, DIGS_20 and DIGS_ALL and are shown in Figure 2A and B with SMO and NN classifiers and other classifiers in Additional file 2.

Generally, the DIGS model is robust with respect to parameter *NoG*, as in the range of *5* to *20*, classification prediction performance is found to be mostly stable, with some improvement observed between *NoG* 5 and 20. Overall, it is noted that prediction performance is case-dependent, not only depending on the dataset under investigation, but also varying with the particular pathway in question (e.g. number of member genes per pathway). In some cases, some improvement is observed against the case of no selection, for example on Yao, Farmer and Pawitan datasets with SMO classifier classification rates increase from 83.7%, 88.3% and 92.9% to 89.5%, 97.6% and 98.8% (*NoG = 5*) respectively (Figure 2A).

The model performs well even in the case where the number of genes is not reduced (see DIGS_ALL in Figures 2 and Additional file 2), indicating that, although reducing the total number of genes per pathway through parameter *NoG* may be beneficial to a particular application, it is by no means compulsory. Therefore, *NoG* offers the *flexibility* of feature reduction, if looking into the effect of a subset of genes is desired, without imposing any additional limitations that would stem from cases where parameter specification would be mandatory. For the implementations discussed below, *NoG* equal to a value of ten was chosen as a sensible compromise of the effects discussed above.

### Classification rate comparison across other methods

**Figure 3:**
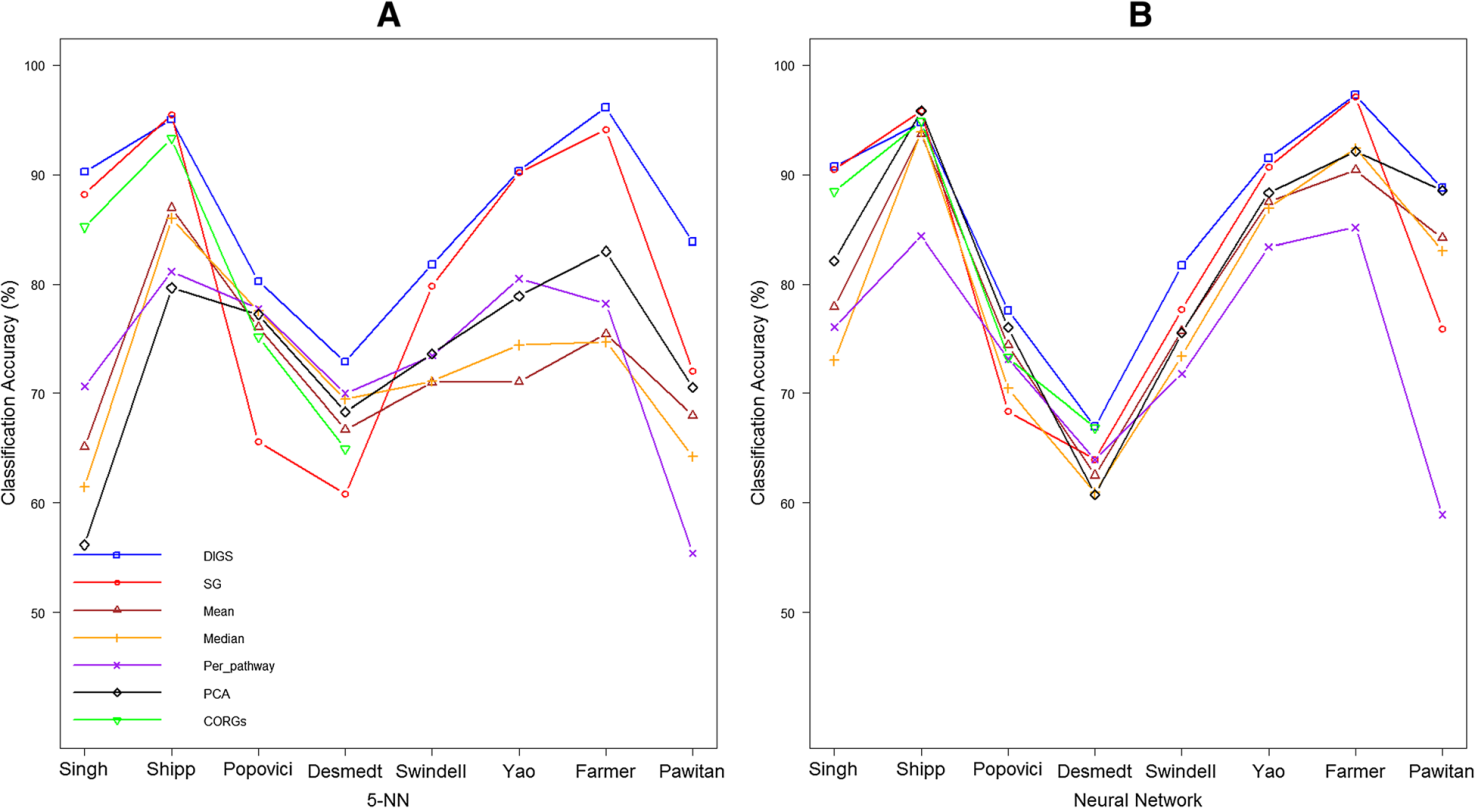
**Classification accuracy comparison of 7 competing methods using 5-NN (A) and NN (B) classifiers.** The proposed DIGS pathway activity inference method is compared against other pathway activity inference methods (Mean, Median, PCA and CORGs) and also genes-based methods (SG and per_pathway). Classification accuracy is summarised as average prediction rates over 50 runs of random partition of datasets into a 70% training set and a 30% testing set. With 5-NN classifier **(A)**, it is evident that DIGS outperforms other methods by some distance as topping the chart on 6 datasets (Singh, Popovici, Desmedt, Swindell, Farmer and Pawitan) while being tied 1^st^ on the other 2 datasets (Shipp and Yao). Prediction rates achieved by DIGS are generally high, over 80% in most datasets, which facilities its application in real world. With NN classifier **(B)**, the same trend can be observed that prediction accuracies achieved by DIGS at least matches the state-of-the-arts methods in literature for binary disease classification problems, while consistently outperforms the competing methods for multi-phenotype problems.

The performance of the proposed DIGS model against other competing methods in literature is compared and discussed here. As described in the Methods section, extensive comparisons were implemented across 8 datasets (collectively referred to as dataset) and 7 competing methods (method). To also account for the effect of classifier choice in the computational procedure, we tested the DIGS model across 5 classifiers (classifier). The results across all dataset, method and classifier combination are illustrated in Figure 3A and B (for 5-NN and Neural Network classifiers) and in the Additional file 3 (for SMO, HB and logistic).

It is obvious from Figure 3A that using 5-NN as classifier DIGS-based classification approach achieves higher classification rates than other pathway activity inference methods, including Mean, Median, PCA, CORGs. On all 8 datasets, DIGS model inferring pathway activity has always outperformed other pathway activity inference methods. It is not a surprise as DIGS seeks to infer pathway activity as of optimal discriminative power. It is also true that DIGS-based pathway activity classification approach results in higher prediction accuracy than Per_pathway, where pathway-specific gene expression profiles are trained and tested independently without constructing pathway activity features. Lastly, the same observation can be made when comparing DIGS to SG, where 186 genes of best discriminative power are selected for classification. DIGS leads to better classification rates than SG on six occasions (Singh, Popovici, Desmedt, Swindell, Farmer and Pawitan), while being tied with SG on Yao and trailing SG by marginal extent on Shipp. Overall it is evident that the proposed DIGS-based classification approach leads to more robust and accurate classification than other state-of-the-arts approaches in literature.

With regards to the actual prediction rates, the combination of DIGS model inferring pathway activity and 5-NN classifier offers prediction rates of above 90% for 4 out of 8 employed datasets, including Singh, Shipp, Yao, Farmer, around 80% for another 3 datasets, including Popovici, Swindell and Pawitan, while still managed 70% for the last dataset Desmedt. The generally high prediction rates demonstrate the applicability and efficiency of the proposed DIGS model in practice.

To show that the desirable prediction rates achieved by DIGS-based approach is not due to a specific bias of DIGS model with 5-NN classifier, we present the classification accuracy comparison using Neural Network classifiers in Figure 3B. According to Figure 3B, when employing Neural Network classifier, DIGS-based disease classification approach again shows great competitiveness in 4 binary datasets that it gives the highest classification rate in Popovici; is tied as the top method in Singh with single genes-based approach and in Desmedt with CORGs; in Shipp DIGS trails the most accurate approach only marginally. In terms of 4 multiclass datasets, DIGS-based classification approach dominates in all of them. The same phenomenon can be observed using the other 3 implemented classifiers that DIGS model either provides competitive classification accuracies or gives the highest classification rate (See Additional file 3 for more details).

To obtain an overview of how our methodology compares across all combinations of dataset, method and classifier, we used a simple normalisation procedure where for each pair of dataset and classifier the actual prediction rates for every method is divided by the highest prediction rates achieved throughout all methods. In other words, the normalised prediction rates, scaled between 0 and 1, reflect the relative performance of a particular method compared against the best performance across all methods for this specific combination of method and classifier. For example, on the Popovici dataset with 5-NN as classifier, the highest prediction rate across all 7 methods (achieved by DIGS as 80.14%) is given a score of 1 and for all other methods their prediction rates are divided with the highest prediction rate (in this case for DIGS), to express the relative performance of that method to the best, e.g. raw prediction accuracy of 75.13% achieved by CORGs is normalised to: 75.13%/80.14% = 0.9375. For each combination of method and classifier, normalised prediction rates are averaged over 4 binary datasets and 4 multiclass datasets and are shown in Tables 3 and 4 respectively.

**Table 3:** **Mean normalised classification rates over 4 two-phenotype datasets according to performance**

**Two-class**	**DIGS**	**SG**	**Mean [** 28 **]**	**Median [** 28 **]**	**Per_pathway [** 72 **]**	**PCA [** 45 **]**	**CORGs [** 19 **]**
5-NN	**0.9988**	0.9071	0.8737	0.8751	0.8903	0.8389	0.9371
NN	**0.9973**	0.9584	0.9323	0.9004	0.9041	0.9480	0.9769
SMO	**0.9757**	0.9474	0.9435	0.9225	0.9325	0.9704	0.9645
HB	**0.9835**	0.9730	0.8819	0.8707	0.8547	0.8402	0.9595
Logistic	0.9318	**0.9816**	0.8902	0.8789	0.8632	0.8482	0.9684
Mean	**0.9774**	0.9535	0.9043	0.8895	0.8890	0.8891	0.9613

The highest classification rate achieved across all competing methods is highlighted as bold for each classifier.

**Table 4:** **Mean normalised classification rates over 4 multi-phenotype datasets according to performance**

**Multiclass**	**DIGS**	**SG**	**Mean [** 28 **]**	**Median [** 28 **]**	**Per_pathway [** 72 **]**	**PCA [** 45 **]**
5-NN	**1**	0.9532	0.8126	0.8090	0.8158	0.8696
NN	**1**	0.9488	0.9402	0.9334	0.8322	0.9585
SMO	**1**	0.9335	0.9372	0.9246	0.8521	0.9452
HB	**1**	0.9241	0.7518	0.7639	0.7893	0.8043
Logistic	**1**	0.8290	0.5614	0.5440	0.5589	0.6450
Mean	**1**	0.91772	0.80064	0.79498	0.76966	0.84452

The highest classification rate achieved across all competing methods is highlighted as bold for each classifier.

In terms of binary datasets, Table 3 clearly indicates that DIGS pathway inference model comes at the top of all methods. This is true in the case of most classifiers used and it is only when using with logistic as classifier where DIGS is outperformed by CORGs and SG. For multi-class datasets (Table 4) DIGS is the best method throughout, indicating the strength of our proposed methodology for the most challenging cases where *multiple* outcomes need to be predicted. This highlights that one of the contributions of this work is to design, according to the authors’ best knowledge, the first supervised pathway activity inference method applicable to both binary and multiclass datasets.

### DIGS release significant disease relevant pathways

Besides the high classification rates achieved by the proposed DIGS model, we have also identified a number of breast cancer pathways that may indicate pathway biomarkers. For Pawitan, where around 90% classification rates can be achieved using DIGS with all 5 classifiers, we employed an information gain feature ranking method in WEKA to rank the constructed pathway activities for each random training set. We record 11 pathways that are ranked more than 20 times as the most discriminative. As we have constrained the proposed DIGS model to allow only *10* genes per pathway to participant in pathway activity inference, we further extract for each identified significant pathway the set of constituent genes included in the active genes more than 10 times.

The set of pathways and genes that are found as most discriminant with our method are listed in the Table 5 below. Apart from obvious links to cancer pathways, such as prostate cancer, and other well-known signalling pathways that are known to be deregulated in tumorigenesis (Wnt signalling [80,81]), we note deregulation of nitrogen metabolism that has recently been linked to breast cancer [82,83]. Ubiquitin-mediated proteolysis is also identified, in accordance to previous reports about the importance of this pathway in disease [84] and is linked to poor survival in breast cancer [85]. Glycosylation is also known to be altered in cancer cells where overexpression of large glycoproteins such as mucins has been characterized [86]. Enzymes from the family of GALNT6 and GALNT14 that we have identified were found to be elevated in breast and gastric carcinomas [87]. We also identify the adherens junction complex, that comprises of cadherins and the catenins, is a major adhesion structure in endothelial cells and has been implicated in playing a fundamental role in controlling the transport across the endothelial barrier and in regulating angiogenesis [88] and has been shown to be affected in invasive breast cancer [89].

**Table 5:** **Significant pathways and constituent genes identified by the proposed DIGS model for Pawitan**

**Pathway name**	**Significant constituent genes**
PROSTATE CANCER	EGFR, TCF7L1, GSTP1, PDGFRA, CCNE1, CHUK, PIK3R3, ERBB2, PIK3R1
UBIQUITIN MEDIATED PROTEOLYSIS	UBE2E3, MID1, SKP2, BRCA1, WWP1
WNT SIGNALING PATHWAY	FZD7, SOX17, TCF7L1, SKP1, SFRP1, FZD8
O GLYCAN BIOSYNTHESIS	GALNT3, GALNT7, GALNT11, GALNT6, GCNT3, B4GALT5, GALNT8, C1GALT1, GALNT12, GCNT4, GALNT14, GALNT10, GALNT2, ST3GAL2, GCNT1, ST3GAL1, C1GALT1C1, GALNT1
ADHERENS JUNCTION	EGFR, ERBB2, TCF7L1, TCF7L2, MET, RAC3, SMAD3, MLLT4, RHOA
ERBB SIGNALING PATHWAY	EGFR, NCK2, ERBB2, AKT3, PAK4, EREG, MAPK9, AKT2
NITROGEN METABOLISM	CA12, CA5A, CA9, GLUL, CA3, CA14, CA8, CA7, CA5B, GLUD1, CA2, AMT, CA6, CA1, CTH, GLS2, GLUD2, HAL, CA4, ASNS, CPS1
DORSO VENTRAL AXIS FORMATION	EGFR, NOTCH1, GRB2, MAPK3, NOTCH3, SOS1, CPEB1, PIWIL2 ETS2, MAPK1, NOTCH4, ETV6, PIWIL1, MAP2K1, NOTCH2, SOS2, ETS1, ETV7, KRAS
ENDOMETRIAL CANCER	EGFR, TCF7L1, ERBB2, TCF7L2, MLH1, ELK1, NRAS, AKT3, ARAF, CTNNA2, PIK3CB, AKT2, CCND1, FOXO3, LEF1
NON SMALL CELL LUNG CANCER	EGFR, AKT3, E2F3, ERBB2, BAD, E2F1, RARB, CDKN2A, PLCG2, GRB2, HRAS, MAPK3, PIK3CD, RXRG, TGFA
PANCREATIC CANCER	EGFR, ERBB2, AKT3, CDKN2A, MAPK9, PLD1, RAC3, RALA, CCND1, E2F3, JAK1, PIK3R1

**Figure 4:**
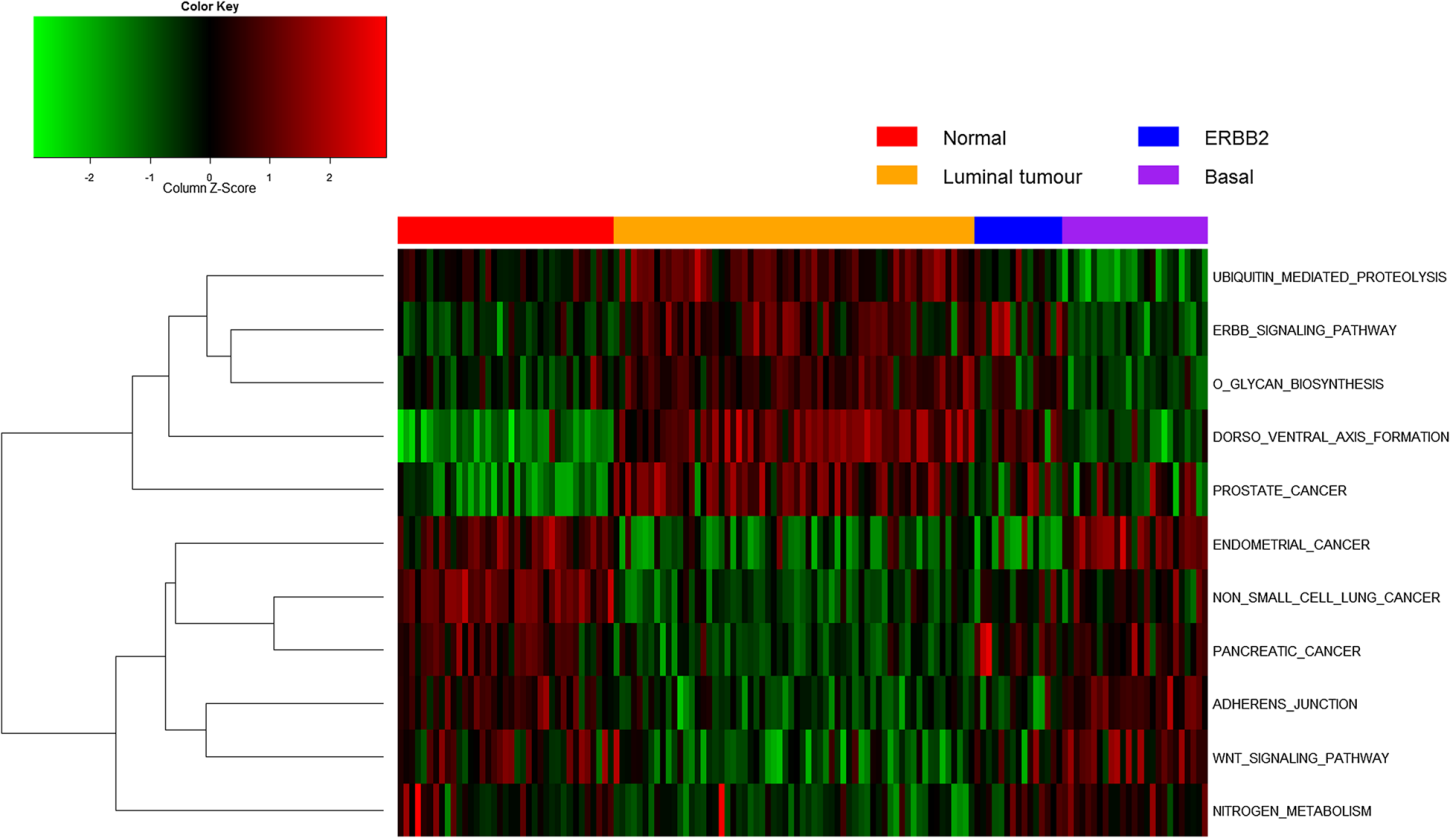
**Pathway activity of the significant pathways in Pawitan.** Pathway activities are inferred with DIGS model using all samples. Red/green blocks indicate up-/down- regulation of pathways (rows) in samples (columns). Pathways are clustered according to similarity of their activities.

We also draw pathway activity heat maps for the significant pathways identified in Pawitan. In Figure 4, pathway activities are inferred using all samples. Pathways are clustered based on similarity of activities on Euclidean distance. It is clear from Figure 4 that pathways are divided into two main clusters, showing distinct patterns of expression. Ubiquitin mediated proteolysis pathway, Erbb signalling pathway, O glycan biosynthesis pathway, Dorso ventral axis formation pathway and prostate cancer pathway are shown to be associated with up-regulation in Luminal tumour, and down-regulation in Basal tumour. The other significant pathways appear to have the opposite regulation mechanism, i.e. they are down-regulated in Luminal tumour and up-regulated in Basal tumours.

We repeat the same analysis of identifying significant pathways and genes for Popovici dataset [8] and Swindell [65] where desirable classification rates can be achieved. The significant pathways/genes and heat map can be accessed at Additional files 4, 5, and 6.

## Conclusions

Incorporating pathway information as biological priors into microarray gene expression profile has been demonstrated to be a promising alternative to conventional genes-based approach in various disease classification problems. However to the authors’ best knowledge there are no supervised pathway activity inference methods for multiclass disease classification problems. In this work, a novel supervised pathway activity inference method for both binary and multiclass disease classification problems, DIGS, has been proposed using mathematical programming optimisation techniques. For each pathway, a new composite feature, called pathway activity, is constructed as a weighted linear summation of expressions of member genes. In each pathway the number of member genes contributing to pathway activity inference by taking non-zero weights is constrained explicitly. The proposed DIGS model provide three main benefits over the existing pathway activity inference methods in literature: (a) the weights of constituent genes in building pathway activity are optimised by DIGS in order to maximise the discriminative power of the pathway activity; (b) the maximum number of constituent genes taking non-zero weights when building pathway activity can be explicitly specified by user; (c) the proposed pathway activity inference model is applicable to both binary and multiclass disease classification problems.

A total number of 8 microarray gene expression profiles totalling 877 samples and ~100,000 genes have been used to demonstrate the applicability and efficiency of the proposed pathway activity inference scheme. The classification results show that for 4 two-class problems DIGS-based classification approaches lead to higher normalised classification performance compared to other existing pathway-based approaches as well as genes-based approaches. In terms of multiclass classification problems, mathematical programming inferring pathway activity here gives consistently the highest prediction accuracies that with the same classifier DIGS always outperforms others by distance.

## Additional files

Additional file 1:
**Notations for mathematical model.**
Additional file 2:
**Sensitivity analysis of parameter** ***NoG*** **for DIGS model with 5-NN (A), HB (B) and Logistic regression (C) classifiers.** For each of the 8 datasets, the proposed DIGS model is applied to infer pathway activity while setting *NoG*, i.e. the maximum number of member genes in a pathway allowed to have non-zero weights, to *5*, *10*, *15* and *20*. In addition, DIGS model is also applied with NoG set to equal to the number of available member genes in a pathway, i.e. all member genes can take non-zero weights to construct pathway activity. A classifier is trained using the pathway activity profiles and tests the prediction accuracy. Additional file 3:
**Classification accuracy comparison of 7 competing methods using SMO (A), HB (B) and Logistic regression (C) classifiers.** The proposed DIGS pathway activity inference method is compared against other pathway activity inference methods (Mean, Median, PCA and CORGs) and also genes-based methods (SG and per_pathway). Classification accuracy is summarised as average prediction rates over 50 runs of random partition of datasets into a 70% training set and a 30% testing set. Additional file 4:
**Significant pathways and constituent genes for Popovici dataset.**
Additional file 5:
**Significant pathways and constituent genes for Swindell dataset.**
Additional file 6:
**Pathway activity of the significant pathways in Swindell dataset.** Pathway activities are inferred with DIGS model using all samples. Red/green blocks indicate up-/down- regulation of pathways (rows) in samples (columns). Pathways are clustered according to similarity of their activities.
