# Supplementary material for: Pathway activity inference for multiclass disease classification through a mathematical programming optimisation framework
Source: BMC Bioinformatics. 2014 Dec 5;15(1):390. doi: 10.1186/s12859-014-0390-2 (PMC4269079; doi:10.1186/s12859-014-0390-2)
Supplement: Additional file 1: — Notations for mathematical model. [file 12859_2014_390_MOESM1_ESM.docx]

| *Indices* |  |
| --- | --- |
| *s* | Sample (*s=1,2,…,S*) |
| *m* | Gene (*m=1,2,…,M*) |
| *c,k* | Class or phenotype (*c=1,2,…,C*) |
| *c_s_* | Class label for sample *s* |
| *Parameter* |  |
| *A_sm_* | Expression level of gene *m* on sample *s* |
| *G_sm_* | Standardised gene expression profile |
| *Ε* | A small positive number |
| *U* | A large positive number |
| *NoG* | Number of member genes allowed to have non-zero weight in building pathway activity for each pathway, a user-specific value |
| *Positive variables* |  |
| *rp_m_* | Positive influence of gene *m* towards pathway activity inference |
| *rn_m_* | Negative influence of gene *m* towards pathway activity inference |
| *Free variables* |  |
| *pa_s_* | Pathway activity of sample *s* |
| *LO_c_* | Lower bound of range of class *c* on pathway activity |
| *UP_c_* | Upper bound of range of class *c* on pathway activity |
| *Binary variables* |  |
| *L_m_* | *1* if effect of gene *m* on pathway activity inference is positive; *0* if negative effect |
| *E_s_* | *1* if pathway activity of sample *s* falls within the range of its class; *0* otherwise |
| *Y_kc_* | *1* if upper bound of pathway activity range for class *k* is lower than lower bound of that for class *c*; *0* otherwise |
| *W_m_* | *1 if gene m is active in pathway activity inference (have non-zero weight); 0 otherwise* |
