# Supplementary figures and images for: Pathway activity inference for multiclass disease classification through a mathematical programming optimisation framework
Source: BMC Bioinformatics. 2014 Dec 5;15(1):390. doi: 10.1186/s12859-014-0390-2 (PMC4269079; doi:10.1186/s12859-014-0390-2)

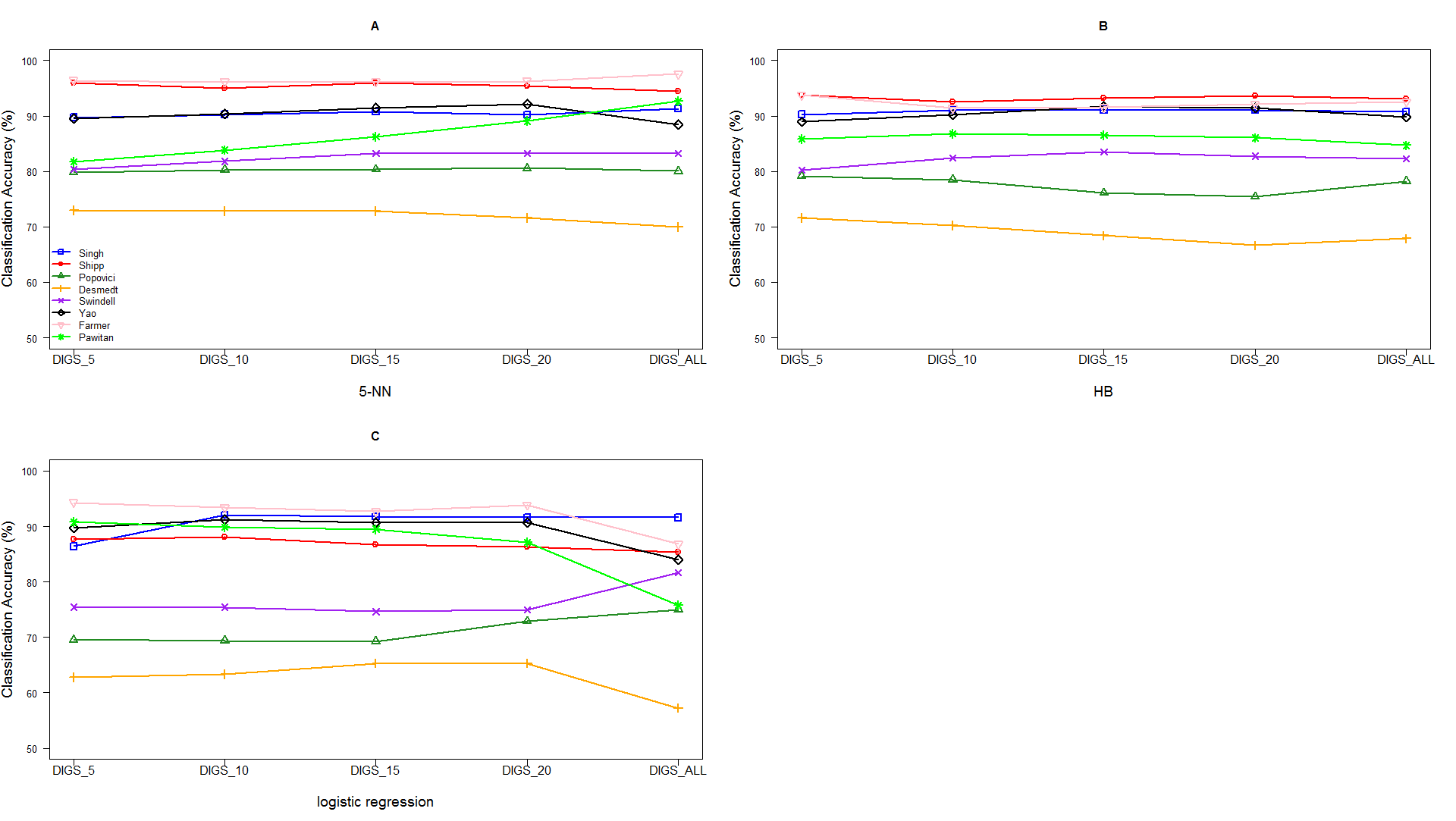

Supplement: Additional file 2: — Sensitivity analysis of parameter NoG for DIGS model with 5-NN (A), HB (B) and Logistic regression (C) classifiers. For each of the 8 datasets, the proposed DIGS model is applied to infer pathway activity while setting NoG, i.e. the maximum number of member genes in a pathway allowed to have non-zero weights, to 5, 10, 15 and 20. In addition, DIGS model is also applied with NoG set to equal to the number of available member genes in a pathway, i.e. all member genes can take non-zero weights to construct pathway activity. A classifier is trained using the pathway activity profiles and tests the prediction accuracy. [file 12859_2014_390_MOESM2_ESM.tiff]

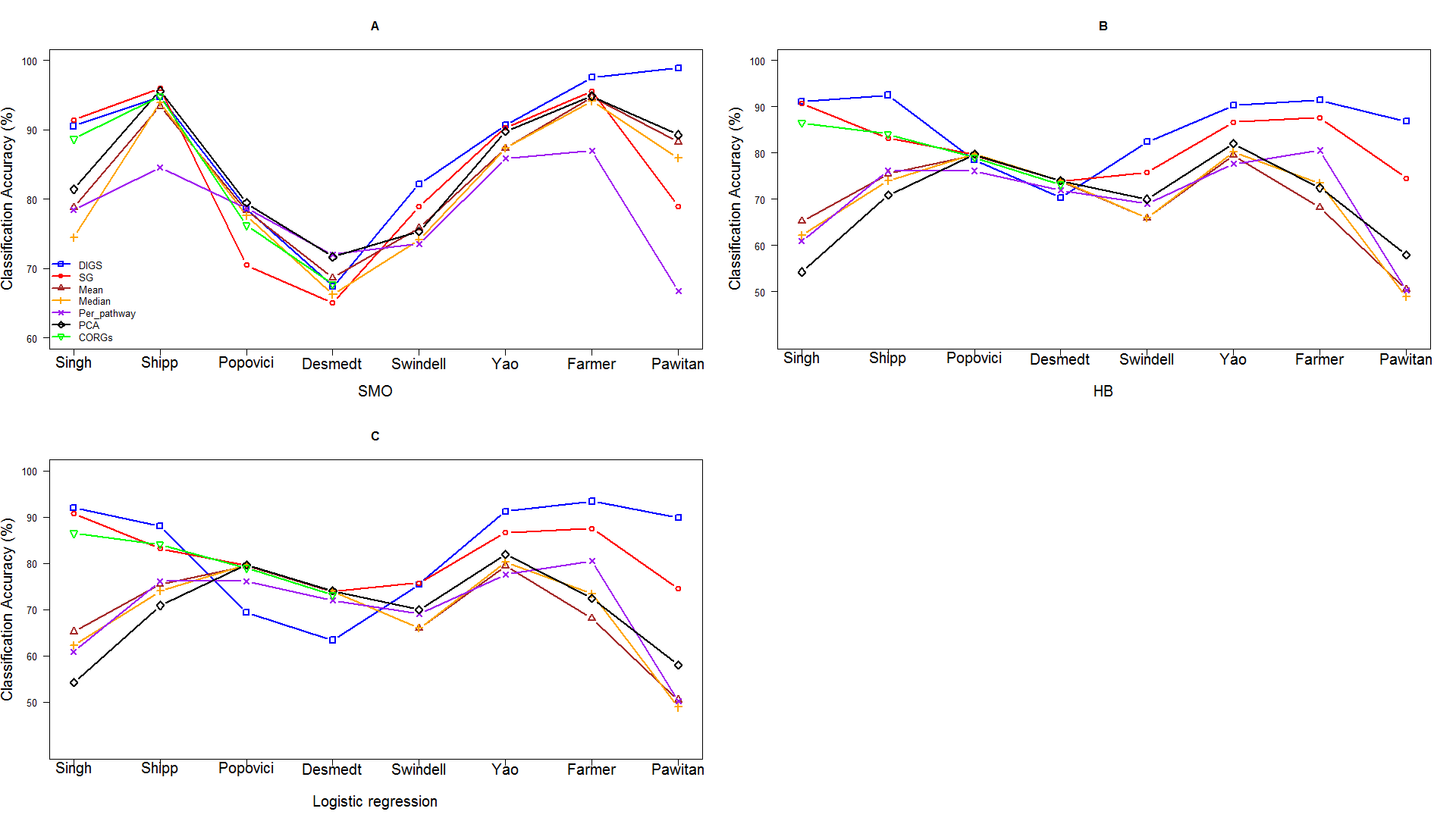

Supplement: Additional file 3: — Classification accuracy comparison of 7 competing methods using SMO (A), HB (B) and Logistic regression (C) classifiers. The proposed DIGS pathway activity inference method is compared against other pathway activity inference methods (Mean, Median, PCA and CORGs) and also genes-based methods (SG and per_pathway). Classification accuracy is summarised as average prediction rates over 50 runs of random partition of datasets into a 70% training set and a 30% testing set. [file 12859_2014_390_MOESM3_ESM.tiff]

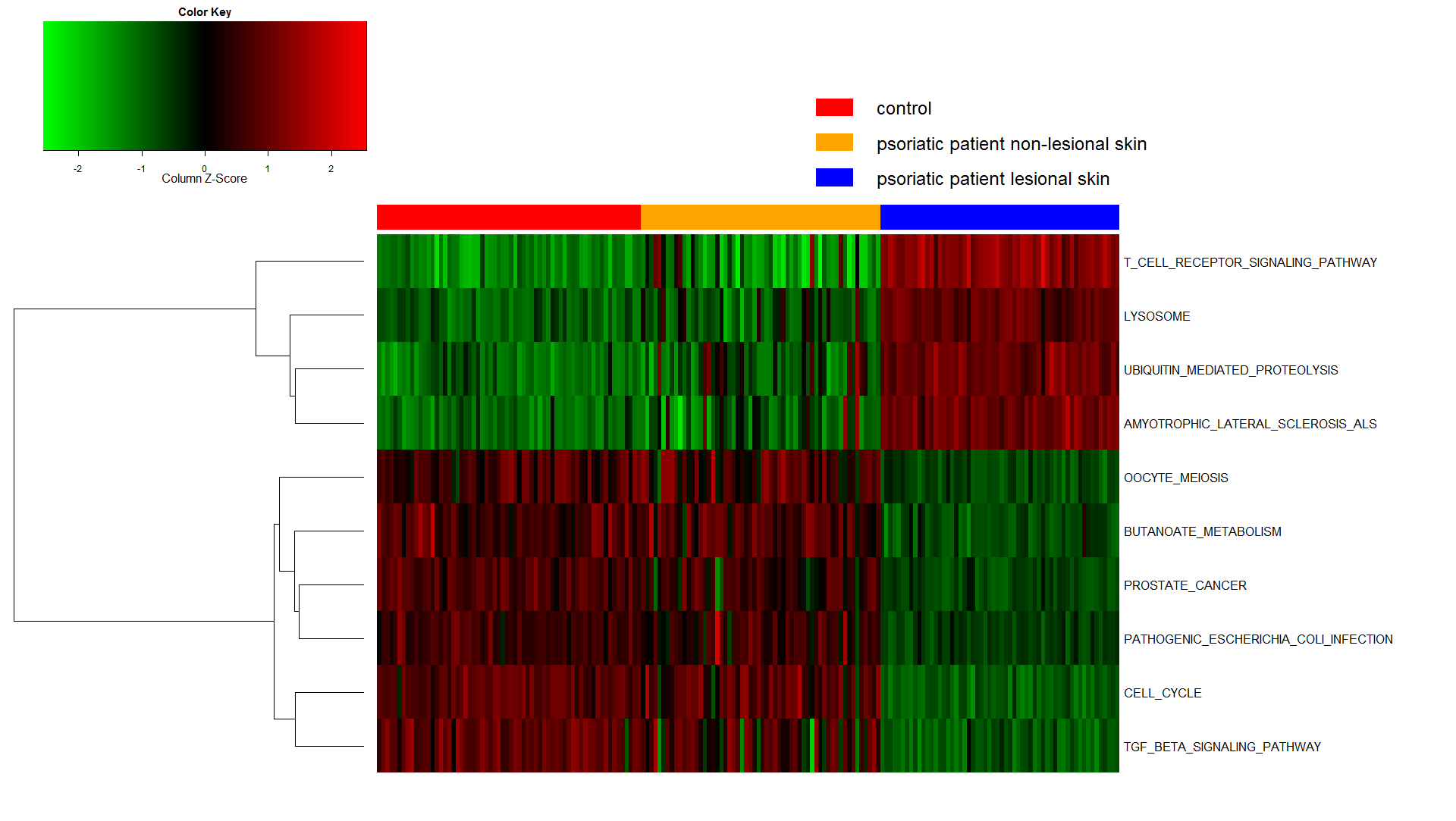

Supplement: Additional file 6: — Pathway activity of the significant pathways in Swindell dataset. Pathway activities are inferred with DIGS model using all samples. Red/green blocks indicate up-/down- regulation of pathways (rows) in samples (columns). Pathways are clustered according to similarity of their activities. [file 12859_2014_390_MOESM6_ESM.tiff]
