# Supplementary material for: Pathway activity inference for multiclass disease classification through a mathematical programming optimisation framework
Source: BMC Bioinformatics. 2014 Dec 5;15(1):390. doi: 10.1186/s12859-014-0390-2 (PMC4269079; doi:10.1186/s12859-014-0390-2)
Supplement: Additional file 4: — Significant pathways and constituent genes for Popovici dataset. [file 12859_2014_390_MOESM4_ESM.docx]

| **Pathway name** | **Significant constituent genes** |
| --- | --- |
| MAPK SIGNALING PATHWAY | DUSP6, MKNK2, PLA2G1B, GADD45G |
| CELL CYCLE | CDK7, CCNH, E2F4, MDM2, TTK, PRKDC, MCM7 |
| GLIOMA | CAMK2B, CALM2, EGFR, E2F3, MAPK3, PIK3R3, CDK4, MDM2, AKT3, CDKN2A, SOS2, GRB2, PIK3CD, CALML5, CAMK2A, PLCG2 |
| BLADDER CANCER | CDKN2A, EGFR, DAPK3, HRAS, DAPK1, MAP2K1, MDM2, CDK4, MAPK1, MMP2, RAF1, MAP2K2, ERBB2, E2F3, DAPK2, FGFR3, E2F1, FIGF, KRAS, PGF, MMP9, THBS1, VEGFA, VEGFB, BRAF, CCND1, CDH1 |
| JAK STAT SIGNALING PATHWAY | IL6ST, PIK3R3, IFNA4 |
| MELANOMA | ARAF, PDGFRB, PIK3R5, AKT3, AKT1, E2F3, EGFR, FGF16 IGF1, MAPK3, BAD, FGF21, PIK3R1, FGF18, CDKN2A, MDM2, AKT2 |
| CHRONIC MYELOID LEUKEMIA | MDM2, IKBKG, E2F3, CCND1, IKBKB, MECOM, HDAC1, BCL2L1, BCR, PIK3R3, CDK4, CTBP2, HDAC2 |
| PANCREATIC CANCER | IKBKG, ERBB2, BCL2L1, AKT3, PIK3R3, EGFR, MAPK3, RALGDS, TGFB3, CDKN2A, E2F3, CHUK, E2F1, EGF, EGFR, JAK1, KRAS, MAPK3 |
| NON SMALL CELL LUNG CANCER | E2F3, ARAF, AKT3, SOS2, BAD, CDK4, PLCG2, PRKCB, CDKN2A, PRKCA, RAF1 |
| NEUROACTIVE LIGAND RECEPTOR INTERACTION | FPR3, HRH2, NPY2R, PRLR |
| PATHWAYS IN CANCER | CTBP1, RBX1, TRAF6 |
| PEROXISOME | ABCD3, ACSL3, DECR2, HAO1, HSD17B4, MPV17, MVK, PAOX, PEX13, PEX5, SCP2 |
| PROSTATE CANCER | CREB3L1, IKBKG, PIK3R3, ERBB2, E2F3, EGFR, GRB2, AKT1, CCNE1 |
