# Supplementary material for: Pathway activity inference for multiclass disease classification through a mathematical programming optimisation framework
Source: BMC Bioinformatics. 2014 Dec 5;15(1):390. doi: 10.1186/s12859-014-0390-2 (PMC4269079; doi:10.1186/s12859-014-0390-2)
Supplement: Additional file 5: — Significant pathways and constituent genes for Swindell dataset. [file 12859_2014_390_MOESM5_ESM.docx]

| **Pathway name** | **Significant constituent genes** |
| --- | --- |
| PATHOGENIC ESCHERICHIA COLI INFECTION | ARPC1A, ARPC3, ARPC5L, CLDN1, CTNNB1, FYN, TUBA1C, TUBA8, TUBB1, TUBB2C, TUBB4, WAS |
| CELL CYCLE | CDC14A, CDK7, DBF4, EP300, GADD45G, HDAC1, MCM5, SKP1,TP53 |
| TGF BETA SIGNALING PATHWAY | ID2, IFNG, INHBC, RBX1, RPS6KB1, SKP1, SMAD9, TNF |
| PROSTATE CANCER | AKT3, CASP9, CCNE1, CCNE2, EP300, INSRR, KRAS, LEF1, PDGFC, PIK3CD, TP53 |
| T CELL RECEPTOR SIGNALING PATHWAY | CHP, CHP2, CHUK, CTLA4, FYN, IFNG, IL2, MALT1, MAP3K14, MAPK13, NCK1, PIK3CD, ZAP70 |
| LYSOSOME | AP3S1, ARSG, ATP6V0C, CTSA, CTSC, CTSS, GM2A, HEXA, HEXB, HGSNAT, LAPTM4A, PSAP, SLC17A5 |
| OOCYTE MEIOSIS | CHP, CHP2, FBXW11, PPP2R1B, RBX1, SKP1, YWHAG |
| UBIQUITIN MEDIATED PROTEOLYSIS | BIRC3, CBL, CUL4A, KLHL13, RBX1, SKP1, SMURF2, UBE3B |
| BUTANOATE METABOLISM | ACADS, ACAT1, ACAT2, ACSM1, ACSM5, AKR1B10, ALDH2, ALDH3A2, ALDH5A1, ALDH7A1, BDH1, ECHS1, EHHADH, GAD1, GAD2, HADHA, HMGCS1, HMGCS2, OXCT1, OXCT2 |
| AMYOTROPHIC LATERAL SCLEROSIS ALS | BAX, BCL2, CASP1, CASP9, CAT, CHP2, GRIA2, GRIN1, MAP2K3, MAPK11, MAPK13, PPP3R2, PRPH2, TNF, TP53 |
